# Resilient and Self-Healing Hyaluronic Acid/Chitosan Hydrogel With Ion Conductivity, Low Water Loss, and Freeze-Tolerance for Flexible and Wearable Strain Sensor

**DOI:** 10.3389/fbioe.2022.837750

**Published:** 2022-02-11

**Authors:** Yunping Hu, Nannan Liu, Kai Chen, Mingxiang Liu, Feng Wang, Pei Liu, Yiyuan Zhang, Tao Zhang, Xiufeng Xiao

**Affiliations:** ^1^ Fujian Provincial Key Laboratory of Advanced Materials Oriented Chemical Engineering, College of Chemistry and Materials Science, Fujian Normal University, Fuzhou, China; ^2^ Fuzhou Second Hospital of Xiamen University, Xiamen University, Fuzhou, China

**Keywords:** hydrogel, self-healing, ionic conductivity, anti-freezing, anti-drying, flexible strain sensor

## Abstract

Conductive hydrogel is a vital candidate for the fabrication of flexible and wearable electric sensors due to its good designability and biocompatibility. These well-designed conductive hydrogel–based flexible strain sensors show great potential in human motion monitoring, artificial skin, brain computer interface (BCI), and so on. However, easy drying and freezing of conductive hydrogels with high water content greatly limited their further application. Herein, we proposed a natural polymer-based conductive hydrogel with excellent mechanical property, low water loss, and freeze-tolerance. The main hydrogel network was formed by the Schiff base reaction between the hydrazide-grafted hyaluronic acid and the oxidized chitosan, and the added KCl worked as the conductive filler. The reversible crosslinking in the prepared hydrogel resulted in its resilience and self-healing feature. At the same time, the synthetic effect of KCl and glycerol endowed our hydrogel with outstanding anti-freezing property, while glycerol also endowed this hydrogel with anti-drying property. When this hydrogel was assembled as a flexible strain sensor, it showed good sensitivity (GF = 2.64), durability, and stability even under cold condition (−37°C).

## Introduction

Hydrogels with a water-rich polymer network structure are very similar to the native tissues of humans (Fergg et al., 2001; Sanchez et al., 2005). In recent years, due to their flexibility, biocompatibility, and designability, hydrogels are widely utilized in various areas, such as tissue engineering (Lee and Mooney, 2001; Khademhosseini and Langer, 2007), wearable devices (Fergg et al., 2001; Yuk et al., 2019; Cui et al., 2021), and flexible electrodes (Na et al., 2019; Zhang W. et al., 2020; Fu et al., 2020). Among these applications, wearable devices, as a new research field, have attracted more and more attention (Billinghurst and Starner, 1999; Seneviratne et al., 2017; Chong et al., 2019). The development of wearable devices provides great convenience for human motion monitoring and human–machine interaction (Liao et al., 2017; Xia et al., 2019; Xu et al., 2019). The hydrogel-based flexible strain sensor was the most commonly used wearable devices, especially for human motion monitoring. First, endowing hydrogels with high conductivity is the foundation for hydrogels working as strain sensors. There are two main solutions: utilization of conductive polymers, such as polyaniline (Guo et al., 2015; Yan et al., 2015) and polypyrrole (Brahim et al., 2002; Shi et al., 2014), and introduction of conductive fillers into hydrogels, such as metal nanoparticles (Guo et al., 2003; Gao et al., 2016), MXene nanosheet (Zhang Y.-Z. et al., 2020, 2021), ionic liquid (Teo et al., 2019), and inorganic salt (Urbonaite et al., 2015; Sui et al., 2021). Various studies have reported that the addition of inorganic salts, such as LiCl, NaCl, KCl, and ZnCl_2_, into the hydrogel systems could bestow the hydrogel with satisfactory ion conductivity (Li et al., 2017, Li et al., 2019, Li et al., 2021; Jiang et al., 2018; Hou et al., 2019). This method is simple, efficient, and economic, showing great potential for conductive hydrogel design and fabrication. On the other hand, the high water content of hydrogels also leads to a serious defect, that is, most hydrogels undergo inevitable freezing when the condition temperature falls below 0°C, the water freezing point (Jing et al., 2013; Bacelar et al., 2016; Mohammadzadeh Pakdel and Peighambardoust, 2018). The hydrogel will become fragile and inelastic at low temperature, which greatly limited the use scenarios of conductive hydrogel as a flexible and wearable strain sensor. Therefore, researchers are deeply aware of the urgency of the development of anti-freezing conductive hydrogels (Tang et al., 2020; Wei et al., 2020; Jian et al., 2021). Actually, freezing point depression is a common phenomenon in nature. For example, seawater still can retain its liquid state at subzero temperature because of the existence of salt. Salt is widely applied as the inhibitor for water freezing in our life, similar to preventing the ice covering of road. Therefore, mixing salt into hydrogels is a simple and convenient strategy to endow hydrogels with conductivity and freeze-tolerance simultaneously (Zhao et al., 2006; Kim T. G. et al., 2008; Jiang et al., 2019; Xu et al., 2021).

Herein, inspired by this mechanism, we designed a natural polymer-based conductive hydrogel with excellent stretchability, self-healing, and anti-freezing property. We choose hyaluronic acid (HA) and chitosan (CS) as the main backbone polymer for our conductive hydrogels due to their good biocompatibility, wide sourcing, and cheap cost (Madihally and Matthew, 1999; Di Martino et al., 2005; Kim I.-Y. et al., 2008, 2011; Burdick and Prestwich, 2011). In order to form reliable crosslinking between HA and chitosan, we first modified HA with hydrazide groups (HA-ADH) and CS with aldehyde groups (OCS). Based on the mild and quick Schiff base reaction between the hydrazide group and the aldehyde group (Cegłowski and Schroeder, 2015; Meher et al., 2018; Xu et al., 2022), the main network of the hydrogel could be rapidly prepared through one-step mixing-injection, while KCl was introduced into this hydrogel system to play the role of conductive and anti-freezing filler. At the same time, we also added glycerol into our hydrogel system which could not only further improve the freeze-tolerance of the hydrogel but also endowed the hydrogel with anti-drying property. This prepared hydrogel named HC-KG hydrogel showed satisfactory mechanical resilience and self-healing property driven by the reversible hydrazone crosslinking, ionic crosslinking, and hydrogen bond–based crosslinking inside the hydrogel. Moreover, our following exploratory experiments showed that this HC-KG hydrogel exhibited repeatable resistance variation responding to various human motions even in the cold when it was applied as a flexible and wearable strain sensor. Therefore, we believe that the exploring of this novel HC-KG hydrogel could further expand the application scenario of conductive hydrogel as a flexible and wearable strain sensor, especially in dry or cold environments.

## Materials and Methods

### Materials

Hyaluronic acid (HA) was purchased from Shandong Focusfreda Biotech Co., Ltd, China. Chitosan (CS), adipic acid dihydrazide, potassium chloride (KCl), glycerol, sodium periodate, ethylene glycol, and N-(3-dimethylaminopropyl)-N′-ethylcarbodiimide hydrochloride (EDC) were purchased from Shanghai Macklin Biochemical Co., Ltd, China. 1-Hydroxybenzotriazole anhydrous (HOBT, 98%) was purchased from J&K. 2-(4-morpholino) ethanesulfonic acid (MES) was brought from Sigma-Aldrich Life Science. All reagents were used as received. Deionized water (DI water) was used for all experiments.

### Preparation of HA-ADH

HA (Mw = 70 kDa) was dissolved in 100 ml MES buffer (PH = 6.5) with the concentration of 1 wt% completely. Then, 2.5 g EDC and 1.78 g HOBT were added into the HA solution carefully. After reaction of 1 h, 9 g ADH was added for another reaction of 24 h. Then, the final solution was dialyzed against DI water (molecular weight cutoff (MWCO) = 14 kDa) for 3 days, and the final product was obtained by freeze-drying at −80°C for 72 h.

### Preparation of OCS

Chitosan (MW = 3 kDa) was completely dissolved in 400 ml glacial acetic acid aqueous solution with a concentration of 0.5 wt%. Then, 2.65 g sodium periodate (NaIO_4_) was added, and the mixed solution was stirred in the dark at 25°C for 12 h. The final solution was dialyzed against DI water (molecular weight cutoff (MWCO) = 14 kDa) for 3 days, and the final product was obtained by freeze-drying at −80°C for 72 h.

### FT-IR Analysis

The successful modification of HA and chitosan was characterized by the FT-IR test using a Bruker Tensor 27 FT-IR spectrometer (ATR-FT-IR, Thermo Fisher Scientific-Nicolet IS5, US). Freeze-dried samples were pressed with KBr and scanned from 4,000 to 500 cm^-1^.

### Preparation of HC Series Hydrogel

Before hydrogel preparation, OCS and HA-ADH solution were prepared with a concentration of 3 and 4%, respectively. Then, the HC series hydrogels were *in situ* formed by mixed injection with HA-ADH and OCS solution. In order to introduce conductive and anti-freeze properties to the HC hydrogel, we added glycerol and KCl to the HC hydrogel. In order to differentiate these HC hydrogels, we abbreviate the HC hydrogel with KCl as HC-K hydrogel, HC hydrogel with glycerol as HC-G hydrogel, and HC hydrogel with both KCl and glycerol as HC-KG hydrogel.

### Rheological Property Test

The rheological properties of the hydrogels were tested at room temperature using a rheometer (Ares G2, TA, and US) with an 8-mm diameter parallel plate and 1-mm gap. Time sweep was performed at fixed frequency of 6 rad/s and strain of 1%. As for the frequency sweep, the strain was fixed at 1%, and the frequency ranged from 0.5 to 500 rad/s. Strain sweep was performed at a fixed frequency of 6 rad/s and a strain range from 1 to 1,000%, and dynamic strain sweeps were performed at a strain range of 1–1,000%. (G′), and loss modulus (G″) of the four hydrogels was measured during the strain sweep.

### Mechanical Test

The mechanical properties of hydrogels were determined by using the universal material testing machine (LR5KPlus, Lloyd, United Kingdom)). For the compression test, a cylindrical hydrogel 12 mm in diameter and 10 mm in height was compressed at a rate of 100 mm/min. In the continuous loading–unloading test, the rate was set at 300 mm/min. The compressive elastic modulus was calculated from the slope of the initial linear region of the stress–strain curve.

### Self-Repair Performance Test

The two pentagram-shaped HC-KG hydrogels were dyed yellow and blue with methyl orange and methylene blue, respectively, and cut into two pieces, and then the two cut hydrogel samples of different colors were closely laminated together and transferred into a well-sealed self-sealing bag at 37°C for 5 min to test the self-healing performance of HC-KG hydrogels.

### Electrical Measurement of Hydrogel

The resistivity of HC, HC-K, HC-G, and HC-KG hydrogels was measured with a digital four-probe tester (Suzhou lattice). The resistance changes of four groups of gel hydrogels were measured by an LCR meter (Changzhou, China). A copper wire is inserted into each end of the hydrogel and then connected it to a TH LCR meter to record the resistance change of the hydrogel in real time. In addition, the resistance change of HC-K and HC-KG hydrogel was measured between −25 and 10°C.

### Fabrication and Testing of the Flexible Sensor

To fabricate the strain sensor, the hydrogel was cut into cylindrical specimens with a diameter of 0.9 cm and a length of 3 cm. Then, the hydrogel sample was assembled into a strain sensor with two layers of conductive copper sheets tightly fixed with copper wires at both ends of the hydrogel sample. The hydrogel was sandwiched between two PU film tapes. To monitor the human motion, the sensor was mounted directly on the skin of the volunteer, and the electrical signal of the strain sensor was recorded in real time using an LCR tester. The simultaneous resistance changes of the gel biosensor under different human motion were obtained by the following equation

$$\frac{\Delta R}{R_{0}}=\frac{R-R_{0}}{R_{0}}.$$

The resistance change ratio (%) was calculated by the following equation, where R_0_ and R are the resistance of the hydrogel when no strain is applied and the resistance of the hydrogel after strain is applied, respectively.

The gauge factor is usually used to characterize the sensitivity of a strain sensor and it defined as

$$GF=\left(\frac{R-R_{0}}{R_{0}}\right)/\left(\frac{L-L_{0}}{L_{0}}\right),$$

where R_0_ and L_0_ are the initial resistance and length of the sensor, respectively, and R and L are the real-time resistance and length of the sensor, respectively.

### Anti-Drying Experiments

The hydrogels were formed into 9-mm diameter cylinders with a volume of 500 μl as test samples and weighed for initial mass. Then, the hydrogel sample was completely exposed to room temperature, and the weight change was recorded according to the time gradient. The residual mass ratio (RMR) was calculated by the following equation:

$$RMR\;=\;\frac{W_{S}}{W_{0}}\times 100\%,$$

where RMR is the residual mass ratio, and W_S_ and W_0_ are the weight of the hydrogel after water loss and the initial weight of the hydrogel, respectively.

### DSC Test

DSC is the study of the heat flow rate versus temperature of a sample and a reference sample by controlling the variation of temperature. We used a differential calorimetry scanner (Mettler Toledo DSC-1, Switzerland) to characterize the hydrogel samples. The samples were encapsulated in a sealed crucible for testing, and an empty disk was used as an inert reference. The differential calorimetry scanner was operated at a nitrogen flow rate of 50 μl/min, and experimental data were recorded at a rate of 1 Hz. Our measurement range is −70–25°C, so the hydrogel samples are first equilibrated at 25°C and then cooled to −70°C at a rate of 5°C/min.

## Results and Discussion

### Synthesis and Characterization of HC-KG Hydrogels

**FIGURE 1:**
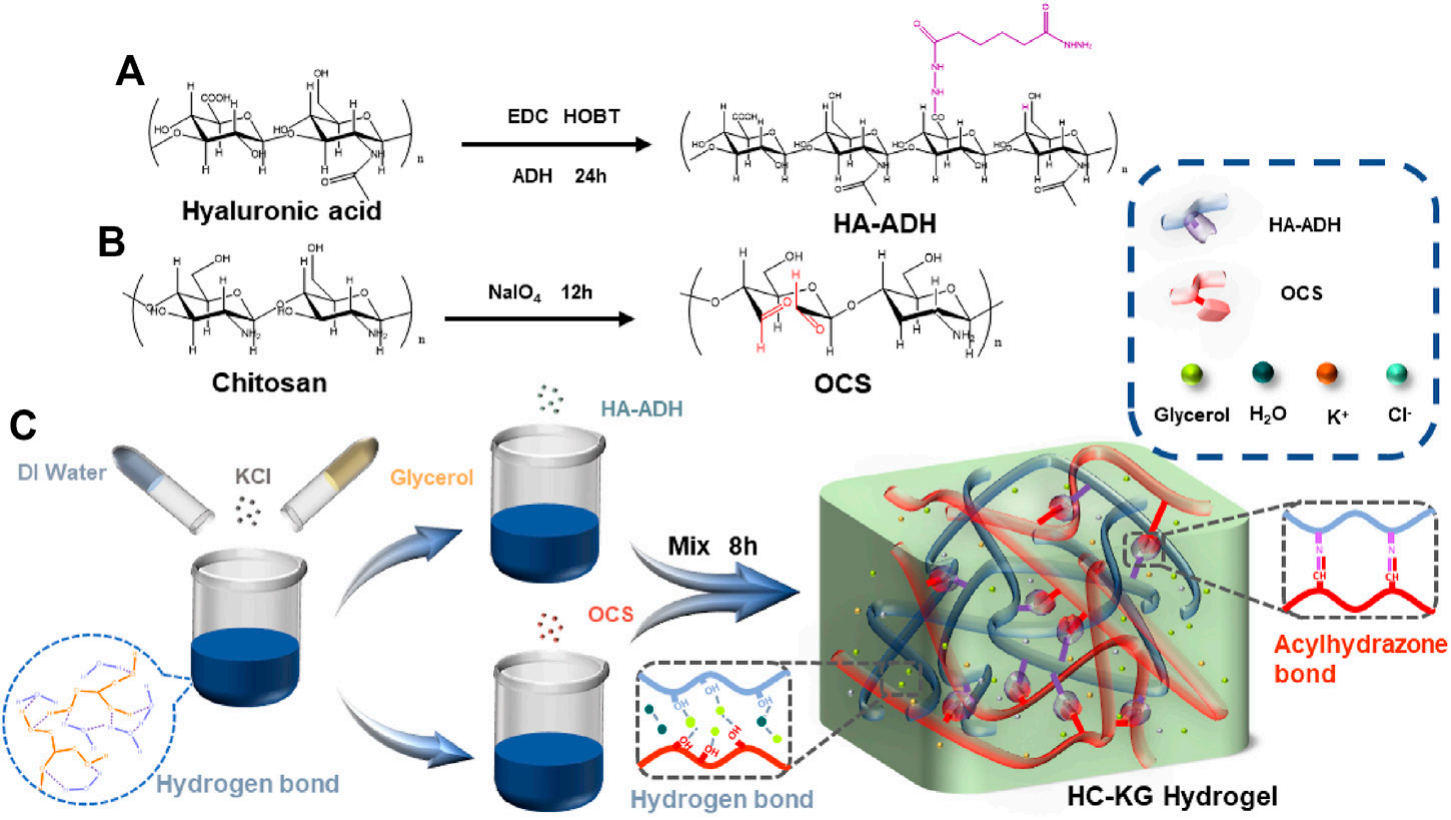
Strategies for the fabrication of the stretchable, self-healing, conductive, and anti-freezing HC-KG hydrogel. The synthesis route of **(A)** HA-ADH and **(B)** OCS **(C)** of the preparation steps and the network structure of the HC-KG hydrogel.

Before the preparation of our well-designed HC-KG hydrogel, we first synthesized the hydrazide-grafted HA (HA-ADH) (Figure 1A) and oxidized chitosan (OCS) (Figure 1B). The Fourier infrared spectrum in Supplementary Figure S1 proved the successful synthesis of these two polymers. The mixed system of KCl/glycerol/water was used as the solvent for HA-ADH and OCS. Then, our conductive and anti-freezing hydrogel, HC-KG hydrogel, was formed by a one-step mixing-injection between the prepared HA-ADH solution and OCS solution with equal volume (Figure 1C), Supplementary Figure S2; Supplementary Table S1 showed that the addition of KCl prolonged gelation time greatly. We speculated that this might be because of the anti-ionization effect (Yang et al., 2020). In detail, K^+^ and Cl^−^ would form reversible ionic crosslinks with negatively charged HA-ADH and positively charged OCS, which led to the intertangling of the HA-ADH, and OCS polymer chain, respectively. Therefore, the steric hindrance of the interaction between HA-ADH and OCS increased.

### Mechanical properties of the HC-KG hydrogel

**FIGURE 2:**
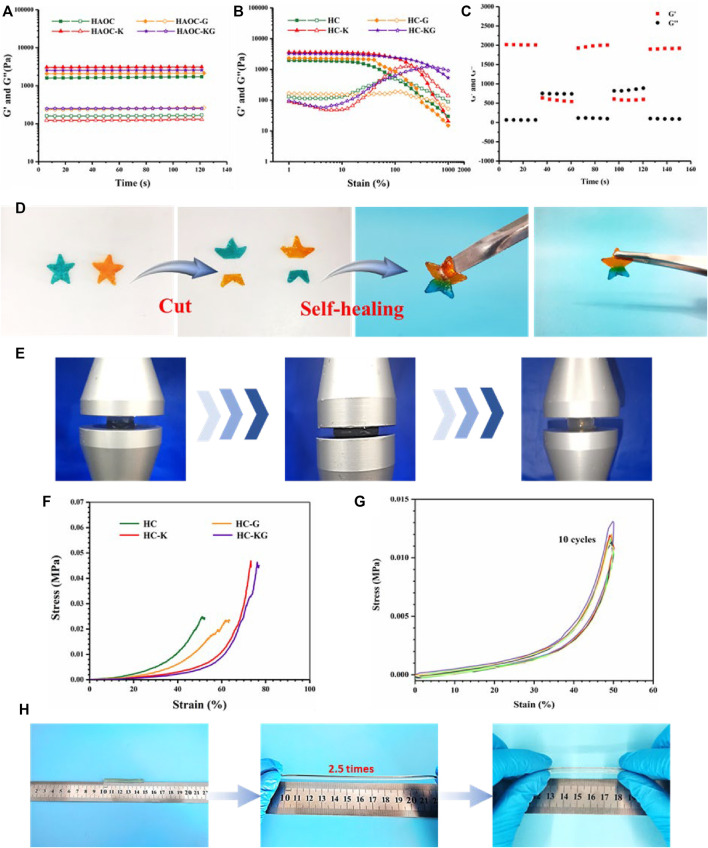
Mechanical properties of the series hydrogel based on HA-ADH and OCS. Rheological time sweep **(A)** and strain sweep **(B)** of HC, HC-K, HC-G, and HC-KG hydrogels. **(C)** Rheological time sweep under alternative switching of low and high strain of the HC-KG hydrogel. **(D)** Quick self-healing property of the HC-KG hydrogel. **(E)** Compression process of the HC-KG hydrogel. **(F)** Compressive strain–stress curve of HC, HC-K, HC-G, and HC-KG hydrogel. **(G)** Cyclic compressive test of the HC-KG hydrogel. **(H)** Stretchability of the HC-KG hydrogel.

We first investigate the mechanical properties of this series hydrogel to investigate the effect of KCl and glycerol on the formation of the hydrogel network. The rheological time sweep results (Figure 2A) demonstrated that the addition of KCl could slightly improve the storage modulus of the hydrogel. We speculated that it was because that K^+^ and Cl^−^ produced by the ionization of KCl helped form extra ionic crosslinking with the negative HA-ADH backbone and the positive OCS backbone, respectively. In addition, glycerol also could improve the storage modulus of the hydrogel. It might be contributed to the newly formed hydrogen bond–based crosslinking among the glycerol and the main backbone of the hydrogel. The further strain sweep results (Figure 2B) also proved our abovementioned speculation. It was clear that the addition of both KCl and glycerol increased the broken strain of the hydrogel. The following time sweep under alternative low/high strain showed the shear thinning property of the HC-KG hydrogel (Figure 2C). In detail, the HC-KG hydrogel would switch to “Sol” state and recover to “Gel” state. This procedure could be repeated many times, and the modulus recovery reached 100% every time. This phenomenon was just because all the crosslinking inside the HC-KG hydrogel network were reversible, including the dynamic hydrazone crosslinking between HA-ADH and OCS; ionic crosslinking between K^+^ and HA-ADH; Cl^−^ and OCS; and hydrogen bonds among glycerol, HA-ADH, and OCS, and these reversible crosslinking could be untied under high shear fore and reformed after the disappearance of the high shear force. Moreover, the abovementioned reversible crosslinking was also the basis of the self-healing property of the HC-KG hydrogel. As shown in Figure 2D; Supplementary Video S1, two individual pentagram-shaped hydrogels, a blue and an orange one, could be reorganized to a new pentagram-shaped hydrogel after being cut. It was based on the re-forming of the reversible crosslinking between the exposed cross-sections. The HC-KG hydrogel also showed excellent compressive and tensile properties. Figure 2E showed that the HC-KG hydrogel had good resilience even under large-scale compression. The quantified compressive strain–stress curve showed that the addition of KCl and glycerol decreased Young’s modulus while increasing the broken strain (Figure 2F). The reason for weakening of Young’s modulus was because the formation of ionic crosslinking introduced by KCl and hydrogen bond introduced by glycerol hindered the formation of the hydrazone crosslinking, and these ionic crosslinking and hydrogen bond were weaker than the hydrazone crosslinking. At the same time, the formation of the weaker ionic crosslinking and hydrogen bond was beneficial to energy diffusion during the compression, therefore increasing the broken strain of the hydrogel. The excellent fatigue resistance was evidently validated by the following cyclic compression test, which showed that the nearly identical repeated strain–stress curves the small hysteresis loop (Figure 2G). The HC-KG hydrogel also showed good stretchability as proven by Figure 2H.

### Conductive Properties of the HC-KG Hydrogel

**FIGURE 3:**
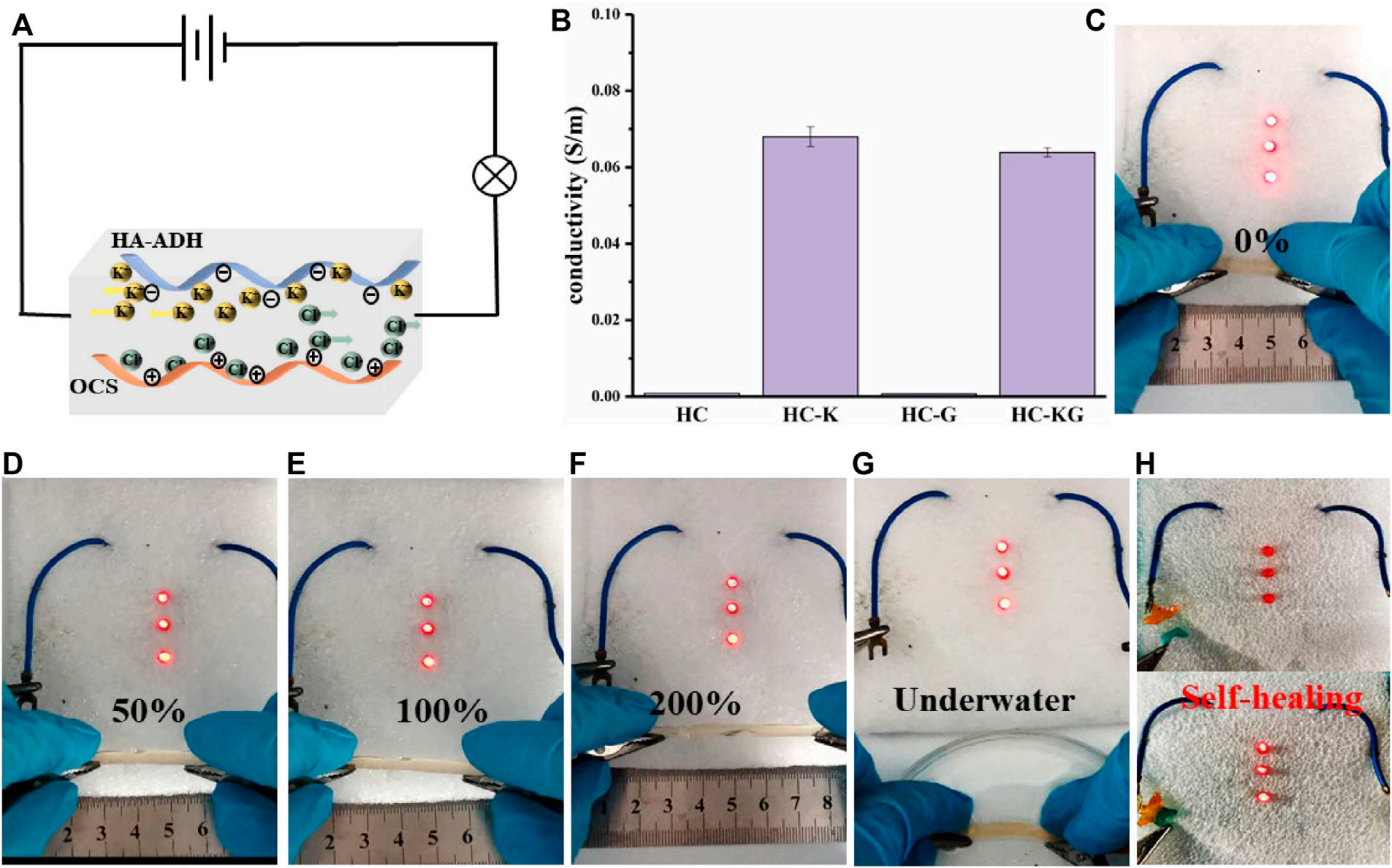
Conductive properties of the series hydrogel based on HA-ADH and OCS. **(A)** Directional movement of ions in hydrogels under electrification. **(B)** Quantitative conductivity of HC, HC-K, HC-G, and HC-KG hydrogel. The HC-KG hydrogel could be applied as a flexible electric wire to lighten the LEDs under different elongation of 0% **(C)**, 50% **(D)**, 100% **(E)**, and 200% **(F)**. **(G)** HC-KG hydrogel also could be applied as a flexible electric wire under water condition. **(H)** HC-KG hydrogel still exhibited good conductivity after its self-healing.

As a strong electrolyte, KCl could fully electrolyze sufficient K^+^ and Cl^−^ after being added into the hydrogel network, thus endowing the HC-K and HC-KG hydrogel with good conductivity, which was strongly proven by Figure 3A. It is also revealed that glycerol had negligible effect on the conductivity introduced by KCl. The conductivity of our target HC-KG hydrogel reached around 0.0638 S/m, which was enough for a flexible strain sensor. We then utilized a long cylindrical HC-KG hydrogel as a flexible electric wire to form a closed circuit to lighten the LEDs. Through the comparison from Figures 3B–F, we could easily find that the brightness of the LEDs decreased gradually with smooth elongation of the HC-KG hydrogel. As an excellent hydrogel strain sensor, HC-KG has good sensitivity with a gauge factor (GF) of 2.64 (Supplementary Figure S3). It indicated the sensitive resistance changing of the HC-KG hydrogel depending on the elongation change, which was fundamental for application of the HC-KG hydrogel as a flexible strain sensor. Furthermore, the HC-KG hydrogel still possessed good conductivity under water condition (Figure 3G), which demonstrated that the application scenario of our HC-KG hydrogel could be expanded to various water environments. Another exciting thing is that the self-healed HC-KG hydrogel still showed satisfactory conductivity (Figure 3H). It implied that the HC-KG hydrogel had the ability to resist various kinds of damage when applied as a flexible strain sensor.

**FIGURE 4:**
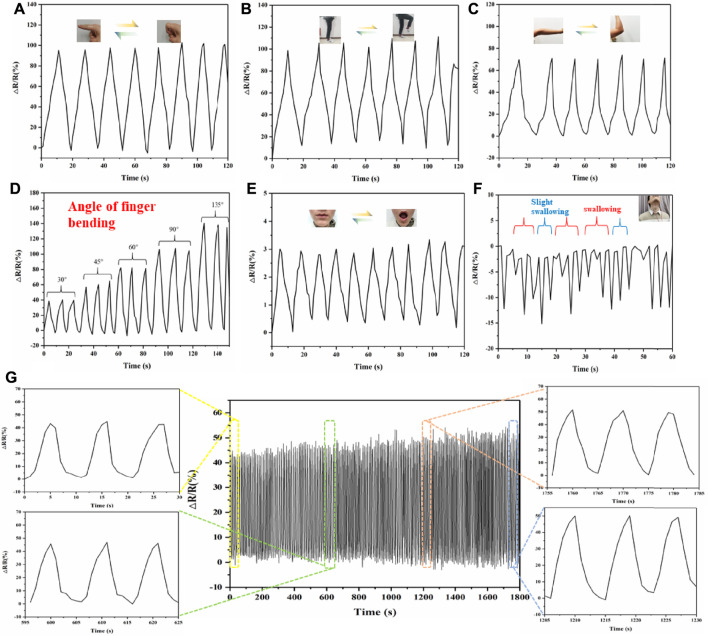
Application of HC-KG hydrogel as a flexible strain sensor to detect various human motions. Real-time monitoring of large human motions, such as the bending of finger **(A)**, elbow **(B)**, and knee **(C)**. **(D)** HC-KG hydrogel showed different relative resistance change for finger bending at different angles. Real-time monitoring of human micromotions, such as the opening and closing of mouth **(E)** and swallowing with different intensity **(F)**. **(G)** Relative resistance change of the HC-KG hydrogel–based strain sensor under cyclic tensile from 0 to 20% for 30 min.

Therefore, we then systematically tested the performance of the HC-KG hydrogel as a flexible and wearable strain sensor. As shown in Figures 4A–C, our HC–KG hydrogel was able to identify the large human motion, including the bending of finger, elbow, and knee. Moreover, taking finger bending as an example, the HC-KG hydrogel could even distinguish different angles of human motion obviously (Figure 4D). Our HC-KG hydrogel could also be competent for the detection of human micromotions, such as the closing and opening of mouth (Figure 4E). In particular, swallowing with different intensity also could be clearly separated by analyzing the curve shape of the relative resistance change (Figure 4F). All of these demonstrated enough sensitivity of our HC-KG hydrogel as the flexible and wearable strain sensor. We monitored the relative resistance change of the HC–KG hydrogel under the cyclic tensile state of 0–20%. The result (Figure 4G) showed that the relative resistance change in every cycle was similar. It strongly confirmed the credible durability and stability of our HC-KG hydrogel, both of which were vital for a satisfactory flexible strain sensor.

### The Anti-Drying and Anti-Freezing Properties of the HC-KG Hydrogel

**FIGURE 5:**
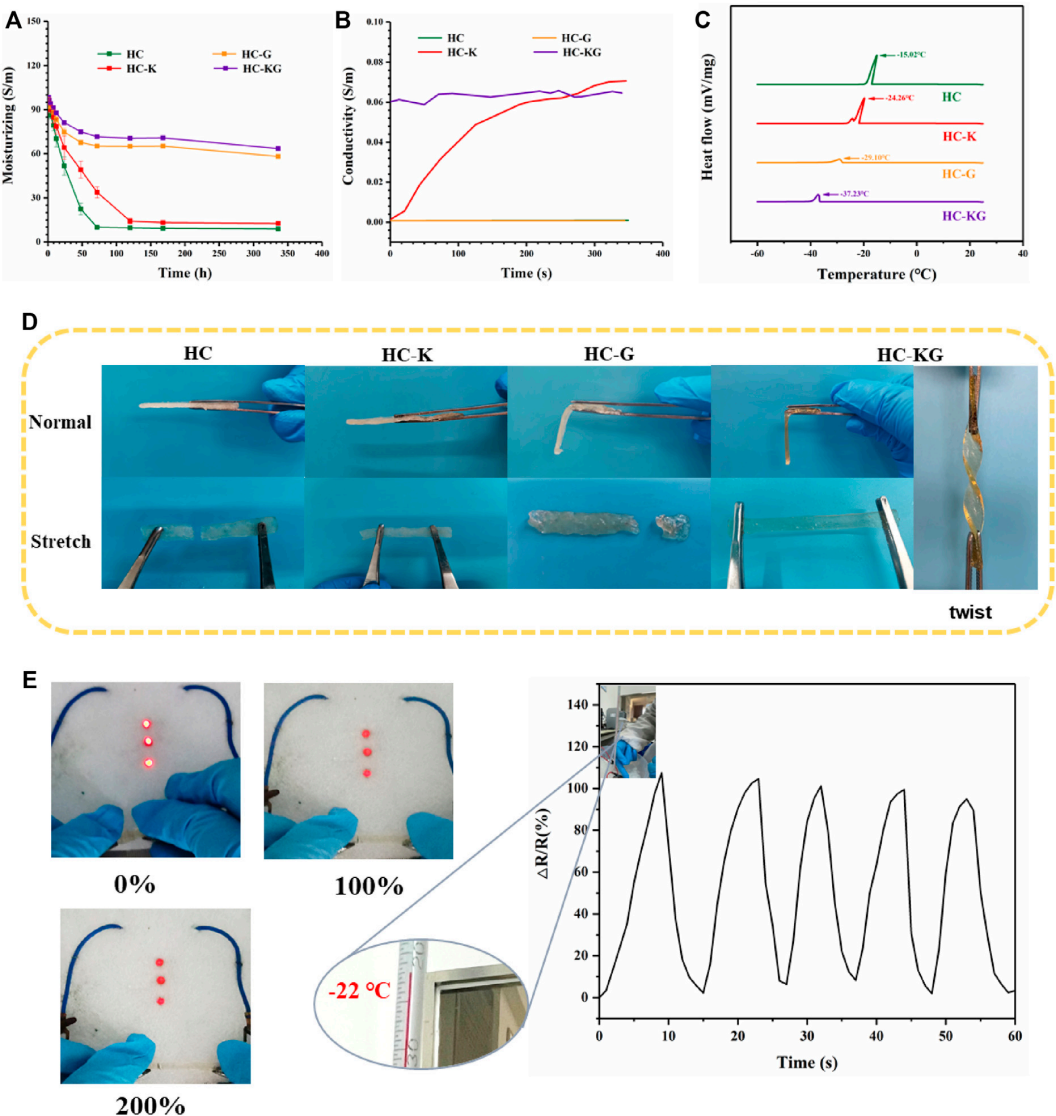
Anti-drying and anti-freezing property of the HC-KG hydrogel. **(A)** Water content changing of HC, HC-K, HC-G, and HC-KG hydrogel under natural condition. **(B)** Conductivity as a function of time after transferring HC, HC-K, HC-G and HC-KG gel from a −26°C refrigerator to a 25°C environment. **(C)** DSC curves of these four kinds of HA-ADH- and OCS-based hydrogel. **(D)** Flexibility comparison of HC-K and HC-KG hydrogel under −26°C. **(E)** Conductivity of the HC-KG hydrogel with different elongation under −26°C. (**F**) HC-KG hydrogel worked as the flexible strain sensor under −20°C.

The high water content of hydrogels also had limited the application of the conductive hydrogel–based flexible strain sensor, which mainly included the following two aspects: 1) The loss of water content of the hydrogel-based sensor led to the instability of their conductivity; 2) The freezing of water inside the hydrogel-based sensor rendered them unable to work at temperatures below 0°C. Our HC-KG hydrogel could solve the abovementioned two problems simultaneously based on the addition of KCl and glycerol. As shown in Figure 5A, hydrogel groups with glycerol (HC-G and HC-KG) could maintain stable water content greater than 60%, while hydrogel groups without glycerol (HC and HC-K) quickly dried up. Actually, the high water loss of the HC-K hydrogel greatly affected its conductivity. Furthermore, the conductivity of HC, HC-K, HC-G, and HC-KG gels was investigated as a function of temperature (Figure 5B). The results showed that the conductivity of HC-KG hydrogels during this process was almost all in the range of 0.63 S/m. While the HC-K hydrogels were more affected by temperature, the conductivity of HC-K hydrogels was almost 0 S/m in the frozen state and then gradually increased to about 0.65 S/m with the increase of temperature. From the differential scanning calorimetry (DSC) results (Figure 5C), we found that the both KCl and glycerol acted as anti-freezing filler when added into the hydrogel system. Among these four groups, the HC-KG hydrogel had the lowest anti-freezing temperature, which reached −37°C based on the synergistic effect of KCl and glycerol. The following Figure 5D; Supplementary Video S2 showed the freeze-tolerance of our HC-KG hydrogel intuitively. At a temperature of −26°C, the HC-KG hydrogel still showed transparency and flexibility. It even could be twisted easily without any damage. The anti-freezing property made our HC-KG hydrogel still suitable to be used as a strain sensor under cold conditions, which was proven by the brightness decrease of LEDs responding to the increase of HC-KG hydrogel elongation under −26°C (Figure 5E). But, the HC-K hydrogel does not conduct electricity at low temperatures to brighten the bulbs (Supplementary Figure S4). We also tried to use this HC-KG hydrogel as a wearable strain sensor to monitor the human motion under cold condition. Taking the monitoring of finger bending as the test model, the HC-KG hydrogel–based wearable strain sensor exhibited good sensitivity (Figure 5F).

## Conclusion

Collectively, the fabrication of our designed HC-KG hydrogel was convenient with low cost and simple process. The biocompatibility of the HC-KG hydrogel was guaranteed by selecting hyaluronic acid, chitosan, KCl, and glycerol as the main raw material. By modifying hyaluronic acid with hydrazide and chitosan with aldehyde, the main network of the HC-KG hydrogel was generated by the reversible dynamic chemical hydrazone crosslinking, which was the product of the Schiff base reaction between hydrazide groups of HA-ADH and aldehyde groups of OCS. Then, the addition of KCl and glycerol would introduce extra ionic crosslinking and hydrogen bond–based crosslinking to the HC-KG hydrogel, and these weak reversible crosslinking bestowed the hydrogel with the ability to withstand great deformation. These reversible crosslinking endowed our HC-KG hydrogel with outstanding self-healing property. Moreover, the HC-KG hydrogel showed good elasticity and fatigue resistance under cyclic deformation. The satisfactory conductivity of the HC-KG hydrogel was contributed by KCl, which was the basis for it to be utilized as the strain sensor. At the same time, the addition of KCl also helped the HC-KG hydrogel resist low temperature condition. Of course, glycerol also contributed greatly to the freeze-tolerance of the HC-KG hydrogel. Based on the synthetic effect of KCl and glycerol, the HC-KG hydrogel even could work at −37°C. Furthermore, glycerol endowed the HC-KG hydrogel with anti-drying property, which guaranteed the unchanged electric property of this hydrogel when it was exposed to outside environment for long time. All the abovementioned characterizations promised sensitivity, durability, and stability of the HC-KG hydrogel during its application as a flexible and wearable strain sensor. Besides, the anti-drying and anti-freezing property expended the application scenarios and working time of the HC-KG hydrogel. In short, this HC-KG hydrogel could be a promising competitor for flexible and wearable strain sensor manufacture, and this study could provide ideas for the following development of flexible and wearable electric sensors.

## Data Availability

The original contributions presented in the study are included in the article/Supplementary Material; further inquiries can be directed to the corresponding authors.

## References

[B1] Bacelar A. H. Silva-Correia J. Oliveira J. M. Reis R. L. (2016). Recent Progress in Gellan Gum Hydrogels provided by Functionalization Strategies. J. Mater. Chem. B 4, 6164–6174. 10.1039/C6TB01488G 32263628

[B2] Billinghurst M. Starner T. (1999). Wearable Devices: New Ways to Manage Information. Computer 32, 57–64. 10.1109/2.738305

[B3] Brahim S. Narinesingh D. Guiseppi-Elie A. (2002). Polypyrrole-hydrogel Composites for the Construction of Clinically Important Biosensors. Biosens. Bioelectron. 17, 53–59. 10.1016/S0956-5663(01)00262-7 11742735

[B4] Burdick J. A. Prestwich G. D. (2011). Hyaluronic Acid Hydrogels for Biomedical Applications. Adv. Mater. 23, H41–H56. 10.1002/adma.201003963 21394792 PMC3730855

[B5] Cegłowski M. Schroeder G. (2015). Preparation of Porous Resin with Schiff Base Chelating Groups for Removal of Heavy Metal Ions from Aqueous Solutions. Chem. Eng. J. 263, 402–411. 10.1016/j.cej.2014.11.047

[B6] Chong Y.-W. Ismail W. Ko K. Lee C.-Y. (2019). Energy Harvesting for Wearable Devices: A Review. IEEE Sensors J. 19, 9047–9062. 10.1109/JSEN.2019.2925638

[B7] Cui C. Fu Q. Meng L. Hao S. Dai R. Yang J. (2021). Recent Progress in Natural Biopolymers Conductive Hydrogels for Flexible Wearable Sensors and Energy Devices: Materials, Structures, and Performance. ACS Appl. Bio Mater. 4, 85–121. 10.1021/acsabm.0c00807 35014278

[B8] Di Martino A. Sittinger M. Risbud M. V. (2005). Chitosan: A Versatile Biopolymer for Orthopaedic Tissue-Engineering. Biomaterials 26, 5983–5990. 10.1016/j.biomaterials.2005.03.016 15894370

[B9] Fergg F. Keil F. J. Quader H. (2001). Investigations of the Microscopic Structure of Poly(vinyl Alcohol) Hydrogels by Confocal Laser Scanning Microscopy. Colloid Polym. Sci. 279, 61–67. 10.1007/s003960000398

[B10] Fu F. Wang J. Zeng H. Yu J. (2020). Functional Conductive Hydrogels for Bioelectronics. ACS Mater. Lett. 2, 1287–1301. 10.1021/acsmaterialslett.0c00309

[B11] Gao Y. Song J. Li S. Elowsky C. Zhou Y. Ducharme S. (2016). Hydrogel Microphones for Stealthy Underwater Listening. Nat. Commun. 7, 12316. 10.1038/ncomms12316 27554792 PMC4999501

[B12] Guo H. He W. Lu Y. Zhang X. (2015). Self-crosslinked Polyaniline Hydrogel Electrodes for Electrochemical Energy Storage. Carbon 92, 133–141. 10.1016/j.carbon.2015.03.062

[B13] Guo H. Hu J.-S. Liang H.-P. Wan L.-J. Bai C.-L. (2003). Highly Dispersed Metal Nanoparticles in Porous Anodic Alumina Films Prepared by a Breathing Process of Polyacrylamide Hydrogel. Chem. Mater. 15, 4332–4336. 10.1021/cm0343397

[B46] Hou W. Sheng N. Zhang X. Luan Z. Qi P. Lin M. (2019). Design of Injectable Agar/NaCl/Polyacrylamide Ionic Hydrogels for High Performance Strain Sensors. Carbohydrate Polymers 211, 322–328. 10.1016/j.carbpol.2019.01.094 30824096

[B14] Jian Y. Handschuh-Wang S. Zhang J. Lu W. Zhou X. Chen T. (2021). Biomimetic Anti-freezing Polymeric Hydrogels: Keeping Soft-Wet Materials Active in Cold Environments. Mater. Horiz. 8, 351–369. 10.1039/D0MH01029D 34821259

[B47] Jiang X. Xiang N. Zhang H. Sun Y. Lin Z. Hou L. (2018). Preparation and Characterization of Poly(Vinyl Alcohol)/Sodium Alginate Hydrogel With High Toughness and Electric Conductivity. Polymer 186, 377–383. 10.1016/j.carbpol.2018.01.061 29456000

[B15] Jiang L. B. Su D. H. Ding S. L. Zhang Q. C. Li Z. F. Chen F. C. (2019). Salt‐Assisted Toughening of Protein Hydrogel with Controlled Degradation for Bone Regeneration. Adv. Funct. Mater. 29, 1901314. 10.1002/adfm.201901314

[B16] Jing G. Wang L. Yu H. Amer W. A. Zhang L. (2013). Recent Progress on Study of Hybrid Hydrogels for Water Treatment. Colloids Surf. A: Physicochemical Eng. Aspects 416, 86–94. 10.1016/j.colsurfa.2012.09.043

[B17] Khademhosseini A. Langer R. (2007). Microengineered Hydrogels for Tissue Engineering. Biomaterials 28, 5087–5092. 10.1016/j.biomaterials.2007.07.021 17707502

[B18] Kim I.-Y. Seo S.-J. Moon H.-S. Yoo M.-K. Park I.-Y. Kim B.-C. (2008a). Chitosan and its Derivatives for Tissue Engineering Applications. Biotechnol. Adv. 26, 1–21. 10.1016/j.biotechadv.2007.07.009 17884325

[B19] Kim I. L. Mauck R. L. Burdick J. A. (2011). Hydrogel Design for Cartilage Tissue Engineering: A Case Study with Hyaluronic Acid. Biomaterials 32, 8771–8782. 10.1016/j.biomaterials.2011.08.073 21903262 PMC3183132

[B20] Kim T. G. Chung H. J. Park T. G. (2008b). Macroporous and Nanofibrous Hyaluronic Acid/collagen Hybrid Scaffold Fabricated by Concurrent Electrospinning and Deposition/leaching of Salt Particles. Acta Biomater. 4, 1611–1619. 10.1016/j.actbio.2008.06.008 18640884

[B21] Lee K. Y. Mooney D. J. (2001). Hydrogels for Tissue Engineering. Chem. Rev. 101, 1869–1880. 10.1021/cr000108x 11710233

[B48] Li H. Lv T. Li N. Yao Y. Liu K. Chen T. (2017). Ultraflexible and Tailorable All-Solid-State Supercapacitors Using Polyacrylamide-Based Hydrogel Electrolyte With High Ionic Conductivity. Nanoscale 9, 18474–18481. 10.1039/C7NR07424G 29159361

[B49] Li J. Wei H. Peng Y. Geng L. Zhu L. Cao X.-Y. (2019). A Multifunctional Self-Healing G-PyB/KCl Hydrogel: Smart Conductive, Rapid Room-Temperature Phase-Selective Gelation, and Ultrasensitive Detection of Alpha-Fetoprotein. Chemical Communications 55, 7922–7925. 10.1039/C9CC02770J 31215917

[B50] Li G. Li C. Li G. Yu D. Song Z. Wang H. (2021). Development of Conductive Hydrogels for Fabricating Flexible Strain Sensors. Small, 2101518. 10.1002/smll.202101518 34658130

[B22] Liao M. Wan P. Wen J. Gong M. Wu X. Wang Y. (2017). Wearable, Healable, and Adhesive Epidermal Sensors Assembled from Mussel-Inspired Conductive Hybrid Hydrogel Framework. Adv. Funct. Mater. 27, 1703852. 10.1002/adfm.201703852

[B23] Madihally S. V. Matthew H. W. T. (1999). Porous Chitosan Scaffolds for Tissue Engineering. Biomaterials 20, 1133–1142. 10.1016/S0142-9612(99)00011-3 10382829

[B24] Meher N. Panda S. Kumar S. Iyer P. K. (2018). Aldehyde Group Driven Aggregation-Induced Enhanced Emission in Naphthalimides and its Application for Ultradetection of Hydrazine on Multiple Platforms. Chem. Sci. 9, 3978–3985. 10.1039/C8SC00643A 29862002 PMC5944821

[B25] Mohammadzadeh Pakdel P. Peighambardoust S. J. (2018). Review on Recent Progress in Chitosan-Based Hydrogels for Wastewater Treatment Application. Carbohydr. Polym. 201, 264–279. 10.1016/j.carbpol.2018.08.070 30241819

[B26] Na R. Liu Y. Lu N. Zhang S. Liu F. Wang G. (2019). Mechanically Robust Hydrophobic Association Hydrogel Electrolyte with Efficient Ionic Transport for Flexible Supercapacitors. Chem. Eng. J. 374, 738–747. 10.1016/j.cej.2019.06.004

[B27] Sanchez C. Arribart H. Giraud Guille M. M. (2005). Biomimetism and Bioinspiration as Tools for the Design of Innovative Materials and Systems. Nat. Mater 4, 277–288. 10.1038/nmat1339 15875305

[B28] Seneviratne S. Hu Y. Nguyen T. Lan G. Khalifa S. Thilakarathna K. (2017). A Survey of Wearable Devices and Challenges. IEEE Commun. Surv. Tutorials 19, 2573–2620. 10.1109/COMST.2017.2731979

[B29] Shi Y. Pan L. Liu B. Wang Y. Cui Y. Bao Z. (2014). Nanostructured Conductive Polypyrrole Hydrogels as High-Performance, Flexible Supercapacitor Electrodes. J. Mater. Chem. A. 2, 6086–6091. 10.1039/C4TA00484A

[B30] Sui X. Guo H. Cai C. Li Q. Wen C. Zhang X. (2021). Ionic Conductive Hydrogels with Long-Lasting Antifreezing, Water Retention and Self-Regeneration Abilities. Chem. Eng. J. 419, 129478. 10.1016/j.cej.2021.129478

[B31] Tang L. Wu S. Qu J. Gong L. Tang J. (2020). A Review of Conductive Hydrogel Used in Flexible Strain Sensor. Materials 13, 3947. 10.3390/ma13183947 PMC7560041 32906652

[B32] Teo M. Y. RaviChandran N. Kim N. Kee S. Stuart L. Aw K. C. (2019). Direct Patterning of Highly Conductive PEDOT:PSS/Ionic Liquid Hydrogel via Microreactive Inkjet Printing. ACS Appl. Mater. Inter. 11, 37069–37076. 10.1021/acsami.9b12069 31533420

[B33] Urbonaite S. Poux T. Novák P. (2015). Progress towards Commercially Viable Li-S Battery Cells. Adv. Energ. Mater. 5, 1500118. 10.1002/aenm.201500118

[B34] Wei P. Chen T. Chen G. Liu H. Mugaanire I. T. Hou K. (2020). Conductive Self-Healing Nanocomposite Hydrogel Skin Sensors with Antifreezing and Thermoresponsive Properties. ACS Appl. Mater. Inter. 12, 3068–3079. 10.1021/acsami.9b20254 31869196

[B35] Xia S. Song S. Jia F. Gao G. (2019). A Flexible, Adhesive and Self-Healable Hydrogel-Based Wearable Strain Sensor for Human Motion and Physiological Signal Monitoring. J. Mater. Chem. B 7, 4638–4648. 10.1039/C9TB01039D 31364689

[B36] Xu G. Guo N. Zhang Q. Wang T. Song P. Xia L. (2022). An Ultrasensitive Surface-Enhanced Raman Scattering Sensor for the Detection of Hydrazine via the Schiff Base Reaction. J. Hazard. Mater. 424, 127303. 10.1016/j.jhazmat.2021.127303 34601405

[B37] Xu J. Wang G. Wu Y. Ren X. Gao G. (2019). Ultrastretchable Wearable Strain and Pressure Sensors Based on Adhesive, Tough, and Self-Healing Hydrogels for Human Motion Monitoring. ACS Appl. Mater. Inter. 11, 25613–25623. 10.1021/acsami.9b08369 31273992

[B38] Xu S. Yan Y. Zhao Y. Qiu X. Zhuang D. Liu H. (2021). Spinnable Adhesive Functional-Hydrogel Fibers for Sensing and Perception Applications. J. Mater. Chem. C 9, 5554–5564. 10.1039/D1TC00151E

[B39] Yan B. Chen Z. Cai L. Chen Z. Fu J. Xu Q. (2015). Fabrication of Polyaniline Hydrogel: Synthesis, Characterization and Adsorption of Methylene Blue. Appl. Surf. Sci. 356, 39–47. 10.1016/j.apsusc.2015.08.024

[B40] Yang J. Shen M. Wu T. Luo Y. Li M. Wen H. (2020). Role of Salt Ions and Molecular Weights on the Formation of Mesona Chinensis Polysaccharide-Chitosan Polyelectrolyte Complex Hydrogel. Food Chem. 333, 127493. 10.1016/j.foodchem.2020.127493 32659659

[B41] Yuk H. Lu B. Zhao X. (2019). Hydrogel Bioelectronics. Chem. Soc. Rev. 48, 1642–1667. 10.1039/C8CS00595H 30474663

[B42] Zhang W. Ma J. Zhang W. Zhang P. He W. Chen J. (2020a). A Multidimensional Nanostructural Design towards Electrochemically Stable and Mechanically strong Hydrogel Electrodes. Nanoscale 12, 6637–6643. 10.1039/D0NR01414A 32175548

[B43] Zhang Y.-Z. El-Demellawi J. K. J. Jiang Q. Ge G. Liang H. Lee K. (2020b). MXene Hydrogels: Fundamentals and Applications. Chem. Soc. Rev. 49, 7229–7251. 10.1039/D0CS00022A 32936169

[B44] Zhang Y. Gong M. Wan P. (2021). MXene Hydrogel for Wearable Electronics. Matter 4, 2655–2658. 10.1016/j.matt.2021.06.041

[B45] Zhao Y. Kang J. Tan T. (2006). Salt-, pH- and Temperature-Responsive Semi-interpenetrating Polymer Network Hydrogel Based on Poly(aspartic Acid) and Poly(acrylic Acid). Polymer 47, 7702–7710. 10.1016/j.polymer.2006.08.056

