# Corrigendum: Resilient and Self-Healing Hyaluronic Acid/Chitosan Hydrogel With Ion Conductivity, Low Water Loss, and Freeze-Tolerance for Flexible and Wearable Strain Sensor

**DOI:** 10.3389/fbioe.2022.880942

**Published:** 2022-04-26

**Authors:** Yunping Hu, Nannan Liu, Kai Chen, Mingxiang Liu, Feng Wang, Pei Liu, Yiyuan Zhang, Tao Zhang, Xiufeng Xiao

**Affiliations:** ^1^ Fujian Provincial Key Laboratory of Advanced Materials Oriented Chemical Engineering, College of Chemistry and Materials Science, Fujian Normal University, Fuzhou, China; ^2^ Fuzhou Second Hospital of Xiamen University, Fuzhou, China

**Keywords:** hydrogel, self-healing, ionic conductivity, anti-freezing, anti-drying, flexible strain sensor

In the published article Nannan Liu was not listed as a co-first author. This has now been rectified.

In addition, there was an error in **affiliation 2**. The name of the hospital just “Fuzhou Second Hospital of Xiamen University” and not “Fuzhou Second Hospital Affiliated to Xiamen University”.

The authors apologize for this error and state that this does not change the scientific conclusions of the article in any way. The original article has been updated.

