# Supplementary material for: Resilient and Self-Healing Hyaluronic Acid/Chitosan Hydrogel With Ion Conductivity, Low Water Loss, and Freeze-Tolerance for Flexible and Wearable Strain Sensor
Source: Front Bioeng Biotechnol. 2022 Feb 11;10:837750. doi: 10.3389/fbioe.2022.837750 (PMC8874126; doi:10.3389/fbioe.2022.837750)
Supplement: Supplementary file 3 [file DataSheet1.docx]

Supplementary Material

**Supplementary Figure 1.** FTIR spectroscopy of HA, HA-ADH, CS and OCS.

In **Figure S1**, Fourier infrared spectrum analysis of CS shows that there was a peak at 3500-3200 cm^-1^ due to the hydrogel action of OH and NH. Compared with CS, the absorption band area of OCS decreased in the range of 3500 ~ 3200 cm^-1^ (-OH and NH) and 2000~2 300 cm^-1^ (H_2_O). This indicates that the free -OH and -NH_2_ were reduced by their oxidation. Amino hydrogel was added to the HA-ADH chain, and there was an absorption peak at 3300-3400 cm^-1^. And the hydroxyl hydrogel on the chain also had an absorption peak at 3200-3650 cm^-1^, so a wide association peak appears, which was the result of the superposition of the stretching vibration peaks of the amino hydrogel and the hydroxyl hydrogel. HA-ADH had one absorption peak at 1650 cm^-1^, which was caused by the stretching vibration of-C=N, while the two absorption peaks at 1607 cm^-1^ and 1560 cm-1 are caused by the stretching vibration of -C=O.


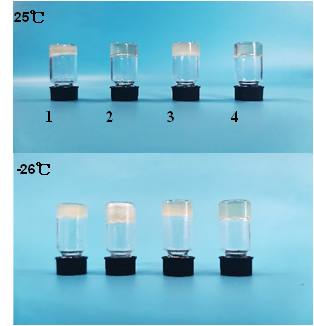


**Supplementary Figure 2.** Schematic diagram of four hydrogels of hydrogel formation and the external appearance at low temperature, 1 for HC hydrogels, 2 for HC-K hydrogels, 3 for HC-G hydrogels, 4 for HC-KG hydrogels.

By mixing 4%HA-ADH and 3%OCS in the binary solution of glycerol/water of KCl (glycerol content is 35%), the two gelled after 8h (**Fig. S2**).


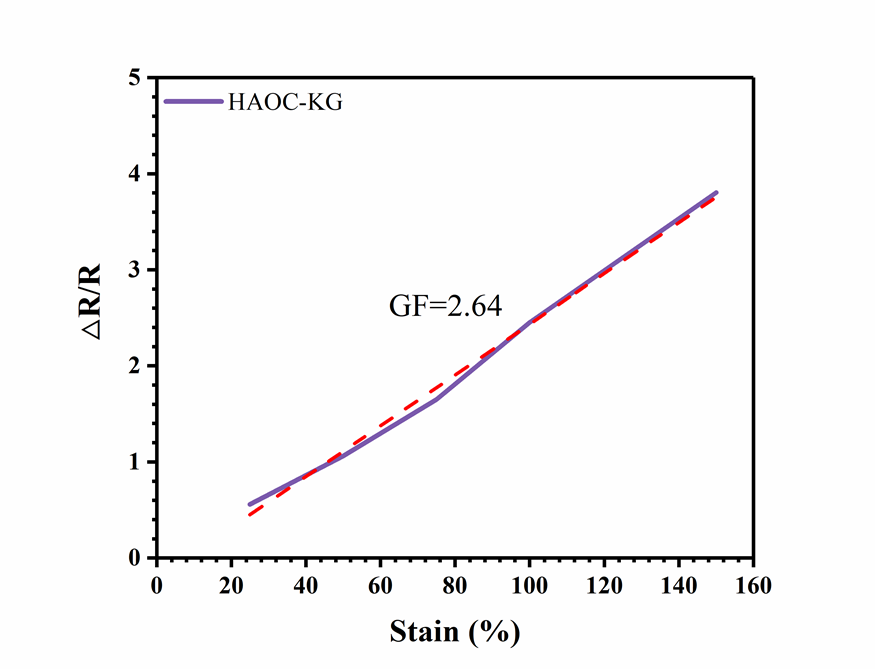
**Supplementary Table 1. Composition and gelation time of hydrogels**

| Hydrogel Name | HA-ADH (wt%) | OCS (wt%) | KCl (wt%) | Glycerin (wt%) | Gel Time |
| --- | --- | --- | --- | --- | --- |
| HC | 4 | 3 | 0 | 0 | 60±3s |
| HC-K | 4 | 3 | 6 | 0 | 7.5h |
| HC-G | 4 | 3 | 0 | 35 | 180±3s |
| HC-KG | 4 | 3 | 6 | 35 | 8h |


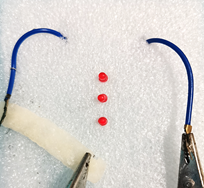
**Supplementary Figure 3.** Relative resistance variations △R/R of HC-KG hydrogel under different tensile strains.

**Supplementary Figure 4.** The HC-K hydrogel is connected to the circuit as a wire at -26°C.
